# Visual Servoing-Based Nanorobotic System for Automated Electrical Characterization of Nanotubes inside SEM

**DOI:** 10.3390/s18041137

**Published:** 2018-04-08

**Authors:** Huiyang Ding, Chaoyang Shi, Li Ma, Zhan Yang, Mingyu Wang, Yaqiong Wang, Tao Chen, Lining Sun, Fukuda Toshio

**Affiliations:** 1 School of Mechatronic Engineering and Automation, Shanghai University, Shanghai 200072, China; dinghyli@163.com; 2 Department of Mechanical and Industrial Engineering, University of Toronto, Toronto, ON M5S 3G8, Canada; chaoyanghit@gmail.com; 3 Provincial Jiangsu Key Laboratory for Advanced Robotics, Soochow University, Suzhou 215123, China; 20165229031@stu.suda.edu.cn (M.W.); 20154229003@stu.suda.edu.cn (Y.W.); chent@suda.edu.cn (T.C.); lnsun@hit.edu.cn (L.S.); 4 Collaborative Innovation Center of Suzhou Nano Science and Technology, Soochow University, Suzhou 215123, China; 5 Intelligent Robotics Institute, School of Mechatronic Engineering, Beijing Institute of Technology, Beijing 100081, China; fukuda@mein.nagoya-u.ac.jp

**Keywords:** automated nanomanipulation, visual servoing, carbon nanotubes (CNTs)

## Abstract

The maneuvering and electrical characterization of nanotubes inside a scanning electron microscope (SEM) has historically been time-consuming and laborious for operators. Before the development of automated nanomanipulation-enabled techniques for the performance of pick-and-place and characterization of nanoobjects, these functions were still incomplete and largely operated manually. In this paper, a dual-probe nanomanipulation system vision-based feedback was demonstrated to automatically perform 3D nanomanipulation tasks, to investigate the electrical characterization of nanotubes. The XY-position of Atomic Force Microscope (AFM) cantilevers and individual carbon nanotubes (CNTs) were precisely recognized via a series of image processing operations. A coarse-to-fine positioning strategy in the Z-direction was applied through the combination of the sharpness-based depth estimation method and the contact-detection method. The use of nanorobotic magnification-regulated speed aided in improving working efficiency and reliability. Additionally, we proposed automated alignment of manipulator axes by visual tracking the movement trajectory of the end effector. The experimental results indicate the system’s capability for automated measurement electrical characterization of CNTs. Furthermore, the automated nanomanipulation system has the potential to be extended to other nanomanipulation tasks.

## 1. Introduction

Carbon nanotubes (CNTs) have drawn a lot of interest over the last decade due to their unique and excellent electrical properties. These superior attributes make CNTs promising candidates for on-chip and off-chip interconnects [1,2]. Recently, there have been many on-going research efforts to develop carbon nanotube-based devices and photodectectors. To realize and broaden these application prospects, plenty of CNT-based materials need to be classified and selected by measuring their electrical attributes through the use of vision-based micro- and nanorobotic systems. However, such characterization procedures remain challenging, because it is extremely difficult to achieve delicate and precise manipulation of CNTs, due to their tiny nanoscaled sizes. Manual nanomanipulation via joysticks is typically applied to control a nanorobotic system to complete these tasks. Such manual operation is time-consuming, inefficient and skill dependent, and the measurement results vary significantly due to poor reproducibility and inconsistency between operators. There is also a steep learning curve, so operators need to undergo a trial-error process to gain skills to avoid damaging either the CNT samples or the fragile end effectors. To address such issues, automated manipulation and measurement tasks that are enabled by visual tracking and servoing techniques are strongly required to achieve more consistent and reliable results as well as batch operation of CNTs.

Research endeavors have been made to achieve semi-automated and fully-automated nanomanipulation inside an SEM to perform precise manipulation and measurement tasks. A high-throughput non-embedded single cell cutting task based on SEM imaging has been performed automatically [3,4,5]. In addition, a dedicated dual-probe setup inside SEM can perform automatically complex alignment sequences to reliably pick-up and release sequences of individual colloidal particles (CPs); this technique is highly promising for complex photonic and/or plasmonic structures consisting of individually sorted CPs with synergistic properties [6,7]. Similarly, automated placement of individual silicon nanowires could be used on a micro electromechanical system device to investigate its nanowires’ electromechanical properties [8]. Fatikow et. al, presented an automated nanohandling workstation for mechanical characterization of nanotubes by measuring Young’s modulus of multiwalled carbon nanotubes (MWCNTs) [9]. Gong et.al implemented visual tracking and servoing control of nanoprobes to perform robust and precise nanoprobing tasks on integrated circuits (IC) [10].

Besides the one or two end-effector nanomanipulation systems mentioned above, increasingly complicated nanomanipulation tasks inside a SEM with multi-manipulators [11,12,13] require an operator to simultaneously manipulate more (more than two) joysticks and/or keypads. Such operations pose practical barriers, because it is difficult to synchronize the control of two manipulators using two hands. Under such circumstances, Yang et al., demonstrated a path planning method to achieve collaborative operation of four nanoprobes under both low and high magnification conditions [14]. In another study, four-point probe measurement of individual nanowires was automatically performed via SEM visual feedback [15].

In regard to target recognition and positioning, template matching [16,17] is a popularly applied visual tracking algorithm for object detection and tracking because of its great applicability. Yet, one major disadvantage of this algorithm is its high computational cost, which results in low update rates using conventional software-based systems. To solve this problem, an advanced method for high-speed template matching based on field programmable gate arrays (FPGA) has been proposed [18,19]. For collaborative operation tasks using multi-manipulators, the sum-of-squared differences (SSDs) algorithm is employed to automatically and simultaneously track four probe tips [15]. Height control is also required to accurately position the delicate nanomanipulation tools but is difficult to achieve due to the lack of Z-direction depth information. To date, a common method of vision-based contact detection has been developed by monitoring the phenomenon that a downward-moving probe slides on the surface of the substrate after contact [8,15,20]. This method has been applied in the detection of the contact between the probe and the nanoparticles [6,7]. However, this contact detection method could cause specimen damage during delicate operations; therefore, a non-contact detection method based on 3D imaging formation has been studied. The 3D images were generated by tilting an electron beam, followed by a stereo algorithm based on a biologically motivated energy model [21]. Otherwise, objects positioned in the Z-direction can be estimated by image sharpness [22,23].

In this paper, we demonstrate a vision-based nanomanipulation system with two Atomic Force Microscope (AFM) cantilevers as the end effectors to perform electrical characterization of carbon nanotubes in a closed-loop control manner. The XY-positions of AFM cantilevers and CNTs are precisely measured via a series of image processing operations. A coarse-to-fine positioning strategy in the Z-direction is applied by the combination of a sharpness-based depth estimation method and a contact-detection method. The use of nanorobotic magnification-regulated speed adapting for automated centering operation of end-effectors is enabled to aid in improving working efficiency and reliability. In addition, we propose automated alignment of manipulator axes by visual tracking the movement trajectory of the end effector. The experiments for automated measurement electrical properties of an individual CNT are performed to validate the effectiveness the proposed algorithms as well as the feasibility of the developed system.

## 2. Automated Nanomanipulation System and Strategy

### 2.1. Nanomanipulation System

**Figure 1:**
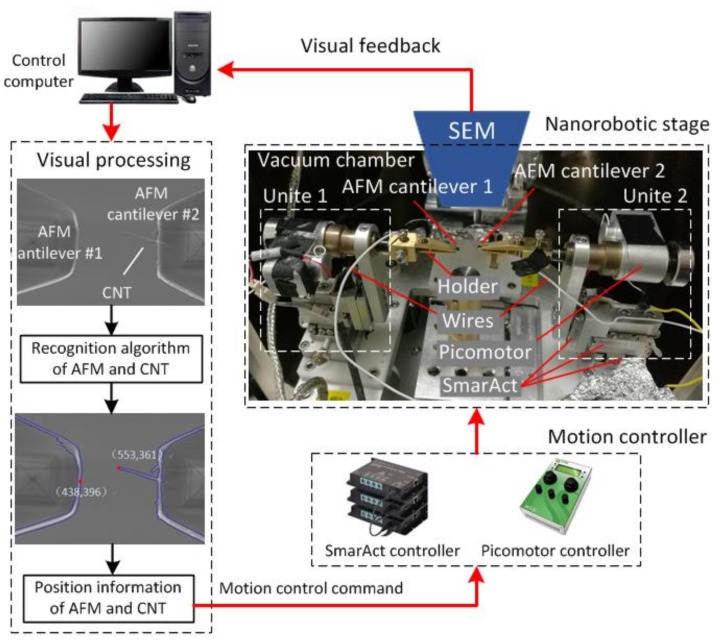
Architecture of nanorobotic manipulation system vision-based feedback.

The nanorobotic manipulation hardware system and the proposed vision-based control scheme are illustrated in Figure 1. This system consists of two nanomanipulator units installed on the specimen stage of an SEM (Merlin Compact, Zeiss, Oberkochen, Germany), two types of piezo motor drivers, an imaging-based guidance system, and a PC. The two nanomanipulator units share the same configuration with 4 degrees-of-freedom (DoFs). Each unit was assembled using three nanomanipulators (SLC-1720, SmarAct, Oldenburg, Germany) to execute linear motions along the X, Y and Z-directions (Size: 22 × 17 × 8.5 ${\mathrm{mm}}^{3}$, travel range: 12 mm, resolution: <1 nm). Another Picomotor (8301-UHV, New Focus Inc., San Jose, CA, USA) was employed and assembled as a rotation actuator to drive the end-effector to rotate along the X axis (Size: 63.5 × 32.2 × 56.5 ${\mathrm{mm}}^{3}$, rotate range: 360-degree, resolution: <1 micro-rad). Two standard silicon nitride AFM cantilevers were respectively attached on the two nanomanipulation units to work as the end effectors. These two cantilevers were utilized to perform the pick-and-place and soldering operations with the EBID technique to assemble a CNT sample, and a CNT adhered to one of two contilevers needed to be measured. Two measuring electrodes (wires) were respectively connected to the two holders of the end effectors to characterize the electrical properties of the CNTs.

The nanorobotic manipulators were controlled by a control PC through controllers (MCS-3D, SmarAct, Oldenburg, Germany and Model 8758, New Focus, CA, USA) to drive the two different types of piezo motors, respectively. The motion control commands for the nanorobot were determined by means of visual feedback based on SEM imaging. The position information of the AFM cantilevers and the CNT tip was detected via the proposed visual detection algorithm and then was sent to controllers to realize closed-loop control in real time.

### 2.2. Overall Strategy of Automated Nanomanipulation

The human operator used joysticks to move the two AFM cantilevers to the field of view (FOV) and then utilized a rotary knob to adjust SEM magnification. When both AFM cantilever tips could be detected in the same FOV with a reasonable magnification, this manual operation process was ended and automated nanomanipulation based on vision feedback commenced.

**Figure 2:**
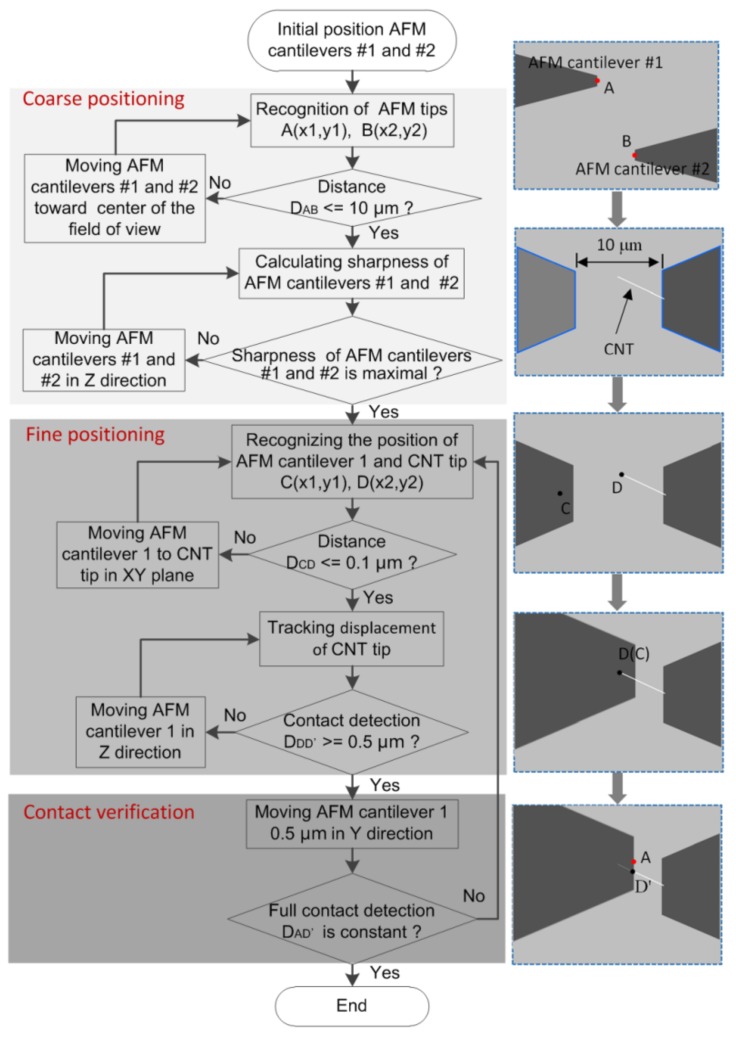
Flow chart for automated measurement on electrical conductivity of an individual CNT.

Because nanomanipulation under a SEM can be precisely performed at a high magnification of at least 3000×, end effectors (AFM cantilevers) needed to be gradually moved to the FOV from a low magnification to a high magnification. However, the process was time consuming, because the operator needed to manually switch the positioner’s speed many times to suit the increasing SEM magnification. Considering this issue, we proposed an efficient coarse-to-fine positioning strategy to automatically characterize the electrical properties of nanotubes. The detailed steps of the automated nanomanipulation process are illustrated in Figure 2.

(1) Coarse positioning stage

For positioning in the XY-plane, the two AFM cantilevers were automatically controlled to move toward the FOV center by visually tracking their tip positions. During the process, the motion speeds of the two AFM cantilevers were decreased with the increase in SEM magnification. When the distance between the center points of the tip positions, denoted as points A and B in Figure 2, decreased to about 10 µm, the coarse positioning was complete.

For positioning in the Z-direction, an approach for sharpness-based depth estimation was developed. The basic idea of this approach was to measure the sharpness of the target object in an image while an observed object was moved in the Z-direction. By tracking the changes in the two cantilevers’ imaging sharpness values, their relative positions in the Z-direction could be roughly estimated.

(2) Fine positioning stage

After completing coarse positioning, the individual CNT soldered onto AFM cantilever #2 could be recognized and localized by image processing. AFM cantilever #1 was visually detected and controlled in the XY-plane to approach the CNT tip from underneath. After point C on AFM #1 coincided with the CNT tip (point D), AFM cantilever #1 was driven along the Z-direction at a low speed to contact the CNT. When AFM cantilever #1 moved close to the CNT, the CNT abruptly deflected downward and adhered on the surface of AFM cantilever #1. This happens due to the increased van der Waals forces between them, and the downwards deflection can be easily detected based on vision feedback. According to this phenomenon, AFM cantilever #1 could be precisely positioned to complete the contact along the Z-direction.

(3) Contact verification

In order to ensure the complete and stable contact, the system automatically monitored whether the contact point (point D’) between AFM cantilever #1 and the CNT was constant. After completing stable contact, the CNT needed to be soldered and fixed on AFM cantilever #1 by electron-beam include deposition (EBID). When the above-mentioned steps were complete, the CNT could be sandwiched between two electrodes of a semiconductor characterization device for further measurement.

## 3. Adopting Methods for Automated Nanomanipulation

### 3.1. Visual Tracking of the CNT Tip and AFM Cantilever

**Figure 3:**
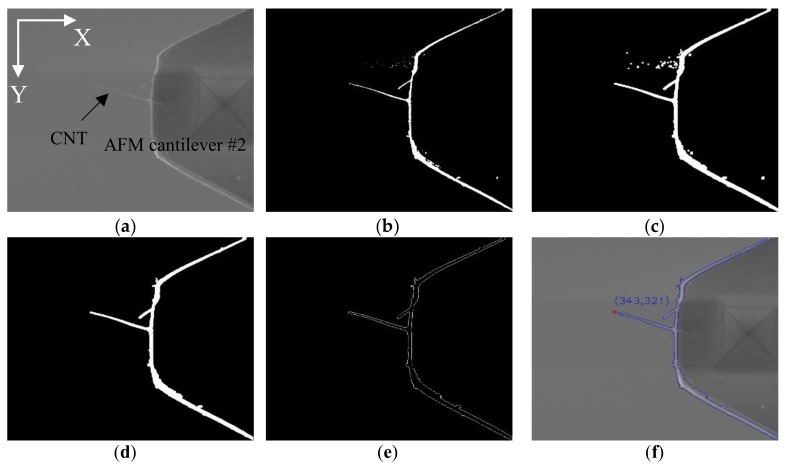
Recognition of CNT tip. (**a**) Raw image; (**b**) Threshold image; (**c**,**d**) Morphological dilation operations; (**e**) Extracting edge contour of AFM cantilever #2 and CNT; (**f**) Finding the CNT tip.

**Figure 4:**
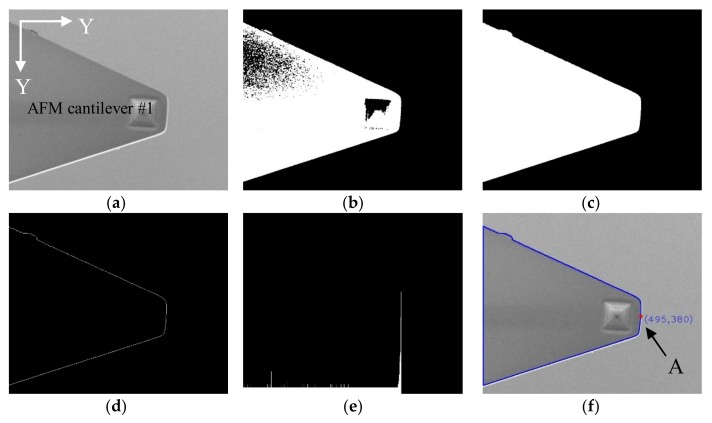
Recognition of AFM cantilever #1. (**a**) Raw image; (**b**) Threshold image; (**c**) Morphological dilation operations; (**d**) Extracting edge contour of AFM cantilever #1; (**e**) Vertical projection of AFM 1 cantilever edge contour along the Y-direction; (**f**) Obtaining a fixed point (A) on AFM cantilever #1.

AFM cantilevers were mounted on nanomanipulators as end effectors to perform nanometer-level operations. A feature-based visual tracking method was conducted to control the movement of the end effectors in the X-Y directions. The original images of the cantilever and the CNT are shown in Figure 3a and Figure 4a, and they were recognized and identified via image processing operations.

There are significant intensity differences among the AMF cantilevers, CNT and background in SEM imaging, facilitating the recognition and extraction of the targeted objects. To overcome the variable brightness and contrast issue and ensure stable extraction, thresholding segmentation, via an adaptive threshold method, was employed to separate AFM cantilever #1 and the CNT from the background. The segmented results were shown in Figure 3b and Figure 4b, and the regions of the AFM cantilevers and the CNT became disconnected. Therefore, morphological dilation operations were followed to modify segmented regions and restore the original shape of the segmented regions, as shown in Figure 3c and Figure 4c. However, the results still contained some small disconnected blobs. To remove them, the area value of each discrete region was calculated, and their values were compared to separate the two maximally-connected regions as the two AFM cantilevers. Small regions were successfully eliminated, as illustrated in Figure 3d. After that, the positions of the CNT tip and AFM cantilever #1 were determined by two different strategies based on their features of edge contour, as shown in Figure 3e and Figure 4d.

The CNT is located on the left side of the used AFM cantilever #2 in the image, and the CNT tip can be detected at the leftmost point of its contour. According to this feature, the successive edge contour can be used to locate the point where the X value was at the minimum, as shown in Figure 3f.

The contour of AFM cantilever #1 can be approximately described as a trapezoid-shaped region; therefore, the above-presented method of CNT tip recognition was not suitable for its determination. A hybrid method was applied via the combination of edge contour projection and a centroid to recognize the position of AFM cantilever #1. Figure 4e shows the vertical projection result of the edge contour along the Y-direction, and a conspicuously leptokurtic histogram can be derived. The X-coordinate with the highest peak value represents the X-coordinate of the cantilever’s right edge. Otherwise, because AFM cantilevers shape was an axial symmetric figure, whose centroid was located on the symmetry line of the AFM cantilevers, the Y-coordinate of the centroid was the same as the midpoint Y-coordinate of AFM cantilever #1’s right edge. The centroid coordinate can be computed via using openCV library functions directly, and the calculating method has been explained in detail in previous literature [14]. Finally, combining the X-coordinate of AFM cantilever #1’s right edge and the Y-coordinate of the centroid, a fixed point (point A) could be determined in real time to describe the position of AFM cantilever #1 (Figure 4f).

### 3.2. Sharpness-Based Depth Estimation

The 2D SEM imaging provided the XY-position of the AFM cantilevers and CNT that can be detected by feature recognition. However, acquiring accurate depth information under SEM imaging is still an intractable issue. The common solutions for obtaining depth information involve capturing multiple images of target objects from different angles by tilting the sample concentrically [24] or the tilting electron beam [21]. The stereo images can be generated using sophisticated algorithms with high calculation cost, resulting in a low frame rate for real-time imaging.

To overcome the above-mentioned issues—complex operations and expensive computational cost—a simpler method that detects the sharpness of a target object around the imaging focal plane has been proposed for depth estimation. The target object was driven to move around the focal plane along the Z-direction. The detected object’s sharpness increased gradually when the object was moved close to the focal plane and achieved its maximum value when it reached the focal plane. This value further declined when the target object continued to move away from the focal plane. By tracking the change of two AFM cantilevers’ sharpness in the image, their positions relative to the focal plane in the Z-direction could be calculated.

**Figure 5:**
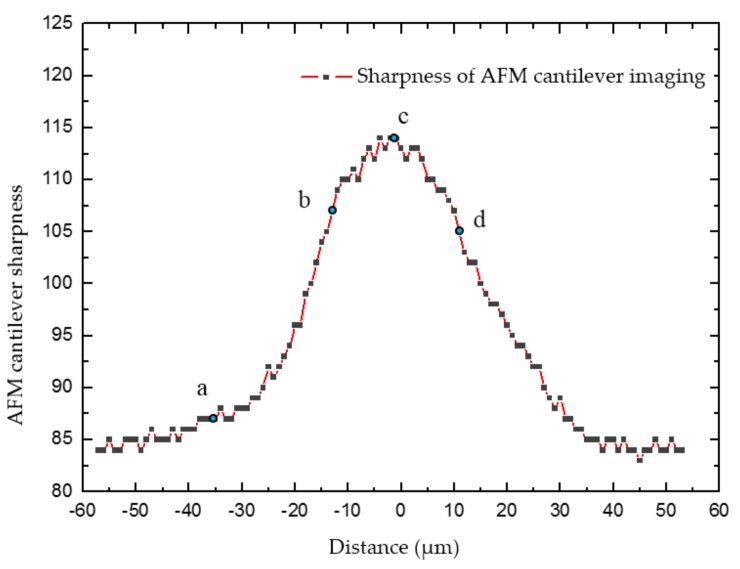
When the AFM cantilevers were moved from underneath to above of the focal plane along the Z-direction at a speed of 2 μm/s, the sharpness of the AFM cantilever image was changed; this was described by the mean gray value of pixels within the AFM cantilever contour.

The focal plane of the SEM imaging was invariant in this work. Therefore, the AFM cantilevers were moved from underneath to above the focal plane along the Z-direction at a speed of 2 μm/s for focal plane search. Meanwhile, the sharpness of AFM cantilevers imaging was estimated by calculating the mean gray value of pixels within the cantilever contour, as shown in Figure 5, and determined by the following Equation (1):

(1) $$\bar{p}=\frac{1}{N}\overset{N}{\underset{i=1}{\sum }}p_{i}\left(x,y\right)$$

where, $\bar{p}$ denotes the mean gray value of pixels within the AFM cantilever contour, $p_{i}\left(x,y\right)$ is the image pixel gray value inside the AFM cantilever contour and *N* expresses the pixel number within the contour.

**Figure 6:**
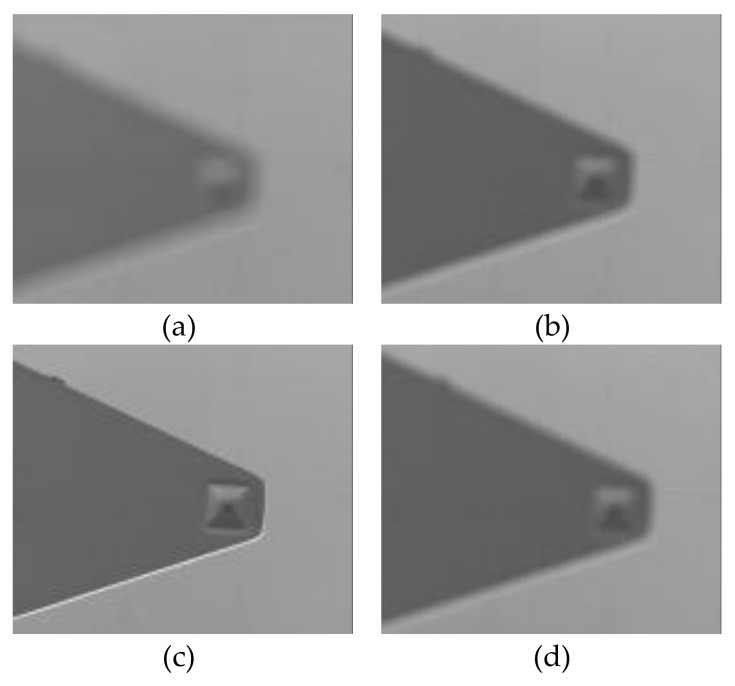
The sharpness variation during the continuous movement of the AFM cantilever in the Z-direction (**a**,**b**) The AFM cantilever located below the focus plane; (**c**) The AFM cantilever reached the focal plane; (**d**) The AFM cantilever located above the focus plane.

The sharpness variation during the continuous movement of the AFM cantilever in the Z-direction, and four typical image sequences are shown in Figure 6. Figure 6a shows the AFM cantilever located below the focus plane; thus, it is defocused with small intensity values. Figure 6b indicates the less defocused state, leading to a higher sharpness compared to Figure 6a. The AFM cantilever was continuously moved upwards and reached the focal plane resulting in a maximum sharpness value, as illustrated in Figure 6c. The corresponding sharpness value decreased in a defocused state when the AFM cantilever was driven away from the focal plane, as displayed in Figure 6d.

After the maximal sharpness value was determined, the corresponding plane was selected as the focal plane. Its Z-direction position was utilized as the origin position, and the other Z-direction movement position values were determined relative to it. The detection accuracy of this depth estimation method is around 3 μm, and it was influenced by the following factors: image noise, image scanning speed and motion speed. Therefore, the sharpness-based depth estimation method was applied for coarse positioning of the AFM cantilevers in the Z-direction. The following physical contact detection between the AFM cantilevers and the CNT was necessary to achieve fine positioning, and a similar approach was implemented in ref. [8]. Through the combination of coarse and fine positioning, the efficiency of automated operation was improved, as well as precisely detecting Z-direction depth.

### 3.3. Magnification-Regulated Speed Adapting Method

For nanomanipulation tasks using SEM imaging of high magnification, most time was devoted to movement of the end effects or simply toward the FOV center, because operators needed to manually switch the positioner’s speed many times to suit different image magnifications. In order to increase the efficiency, a magnification-regulated speed adapting approach was implemented based on visual servoing control.

**Figure 7:**
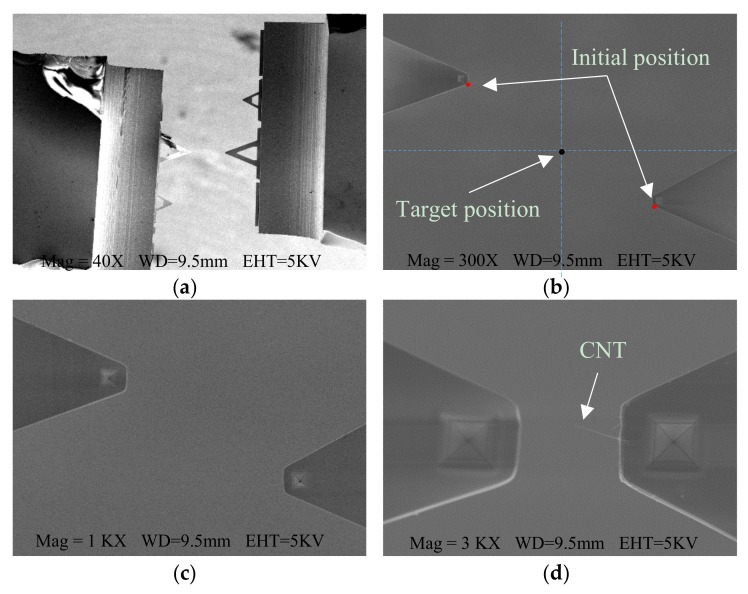
Demonstration of the automated centering procedure of both AFM cantilevers. (**a**) Bringing both AFM cantilevers into the FOV under low magnification; (**b**) Increasing SEM magnification until only the cantilevers’ distal parts can be seen, to start the automated operation; (**c**) Moving the end-effectors to the FOV center for further enlargement; (**d**) The soldered CNT can be detected at an image magnification of 3000×.

Figure 7 depicts the overall procedure that drives both end-effectors to the FOV until the CNT soldered to the distal end of the right AFM cantilever can be detected. At the beginning, the nanomanipulators brought the two AFM cantilevers from both sides into the FOV under low magnification, as shown in Figure 7a. Then the magnification was increased until only the cantilevers’ distal parts could be seen in the FOV as shown in Figure 7b, and the automated centering operation was performed, taking the detected AFM cantilevers’ positions as the initial positions. Thereafter, the operator sent commands using GUI software by visual recognition to automatically move both AFM cantilevers to the FOV center for further enlargement (Figure 7c). When the soldered CNT could be recognized, the SEM magnification value was always no less than 3000×, as shown in Figure 7d.

During the overall movement procedure, the positioner’s movement speed had to be suitable for the current SEM magnification. If the movement speed is too fast at a higher magnification, the targets may move outside the FOV, leading to tracking loss and even collision. On the contrary, if the movement speed is too slow at a lower magnification, this results in inefficiency for performing.

Long-distance nanomanipulation tasks. In addition, selecting a suitable speed using the buttons of hand controllers under different magnification values is time consuming and tedious. Accordingly, a magnification-regulated speed adapting approach for automated centering was proposed. The relationship between the absolute speed of the nanomanipulators and the current imaging magnification can be represented by the following Equation (2):

(2) $$V_{act}=k\times P_{m}=k\times \frac{p_{size}}{M}$$

where, $V_{act}$ denotes the positioner’s absolute movement speed (nm/s), and *k* is a constant which can be selected (*k* = 20, 40 or 60) for setting different speed levels. $P_{m}$ represents the conversion unit between the physical dimension and one pixel under the current magnification; $p_{size}$ expresses the physical size of a pixel which is about 1.116 μm at a magnification of 100×. M is defined as the SEM magnification. The function shows an inverse correlation between SEM magnification and positioner speed. With an increase in SEM magnification, the positioner’s absolute speed ($V_{act}$) decreased, but the relative speed of nanomanipulators in SEM imaging was constant (*k* pixels/second) under any magnification, which can be calculated using Equation (3):

*V_img_* = *M* × *V_act_* = *k* × *p_size_* (3)

where, $V_{img}$ denotes the relative speed of a nanomanipulator in an image which is a constant.

**Figure 8:**
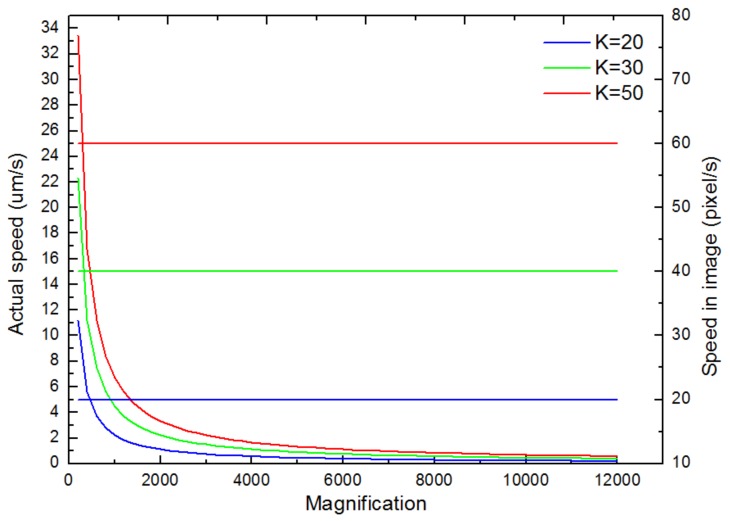
The relationship between the X-axis actual speed and relative speed in SEM imaging with an increase in magnification value, when k was set to different values.

**Figure 9:**
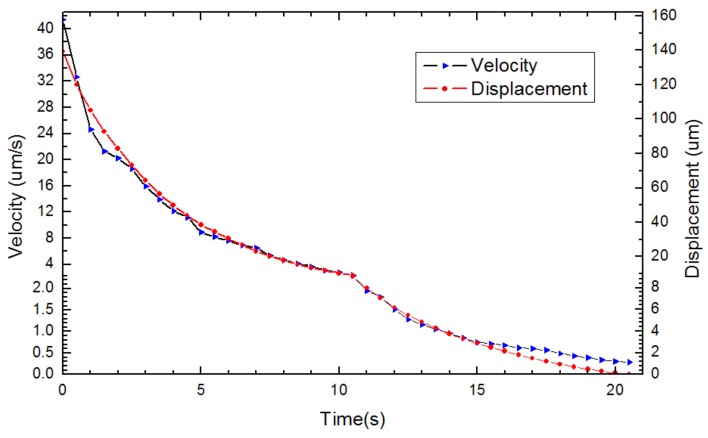
Characterization of automated centering procedure vision-based tracking for the X-axis. Velocity vs. time (black line), displacement vs. time (red line).

With the increase in image magnification, the characterization curves between absolute speed and relative speed in SEM imaging were demonstrated in Figure 8, separately for k values of to be 20, 40 and 60. The relationship between the X-axis velocity and displacement of the AFM cantilever versus time was also determined during the automated centering process (*k* = 40), as depicted in Figure 9. The results indicate that the AFM cantilever moved with a high speed of around 40 μm/s at the beginning, which could improve the working efficiency when it is far from the target position. Then, the AFM cantilever gradually slowed down to a speed of around 500 nm/s. This slow movement can guarantee the positioning accuracy when approaching the target position. The overall movement procedure took approximately 3 min by manual manipulation. Comparatively, this magnification-regulated speed adapting method just needed 21 s, which was nine times less than with manual manipulation by a highly skilled operator. Furthermore, this method maintained higher reliability to avoid the undesired destruction of specimens and nanomanipulators that can result from setting the nanomanipulator at an unsuitable speed.

### 3.4. Vision-Based Alignment of Manipulator Axes and Imaging Axes

**Figure 10:**
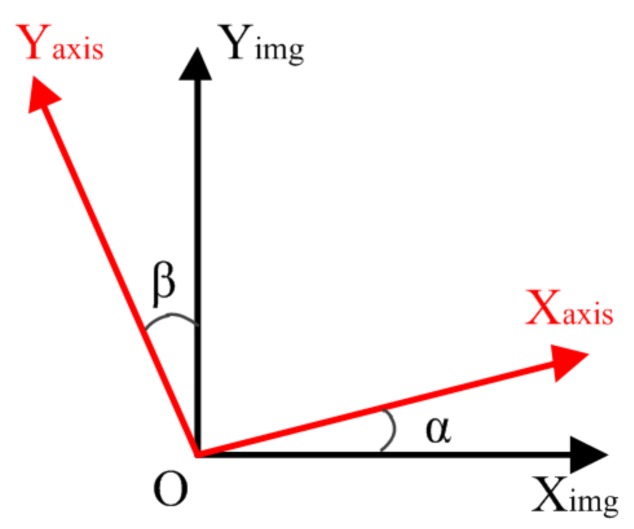
The relationship between the manipulator’s motion axis and the SEM imaging axis.

Due to inaccuracies in the fabrication and assembly of the nanomanipulator components, it was challenging to mechanically align the motion axes of each manipulator and image coordinate axis to be perfectly orthogonal to each other. Therefore, there existed a certain angle between each manipulator’s X/Y-axis and the image’s X/Y-axis, as shown in Figure 10. The intersection angles between the manipulator motion X/Y axis and image X/Y axis are denoted as *α* and *β* for the following derivation, respectively.

The small misalignment angle can induce large motion errors for the closed-loop control of the nanomanipulation system based on vision feedback. Therefore, it was essential to transform the image coordinate system to a motion coordinate system when performing nanomanipulation. To this end, a rotation matrix, *T*, that can realize this coordinate transform was used to correct the misalignments in motion axes, and it can be expressed as follows.

(4) $${\left[\begin{array}{c} x\\ y\\ z \end{array}\right]}_{axis}=T{\left[\begin{array}{c} x\\ y\\ z \end{array}\right]}_{img}=\left[\begin{array}{ccc} \cos\alpha & -sin\beta & 0\\ \sin\alpha & \cos\beta & 0\\ 0 & 0 & 1 \end{array}\right]{\left[\begin{array}{c} x\\ y\\ z \end{array}\right]}_{img}$$

where, ${\left[\begin{array}{ccc} x & y & z \end{array}\right]}_{axis}^{\prime }$ denotes the end-effector’s position in the coordinate system of the motion axes, while ${\left[\begin{array}{ccc} x & y & z \end{array}\right]}_{img}^{\prime }$ represents the end-effector’s position in the image coordinate system. Two-dimensional SEM imaging can be applied to determine this transformation matrix; therefore, the Z-direction position cannot be reflected by this transformation.

**Figure 11:**
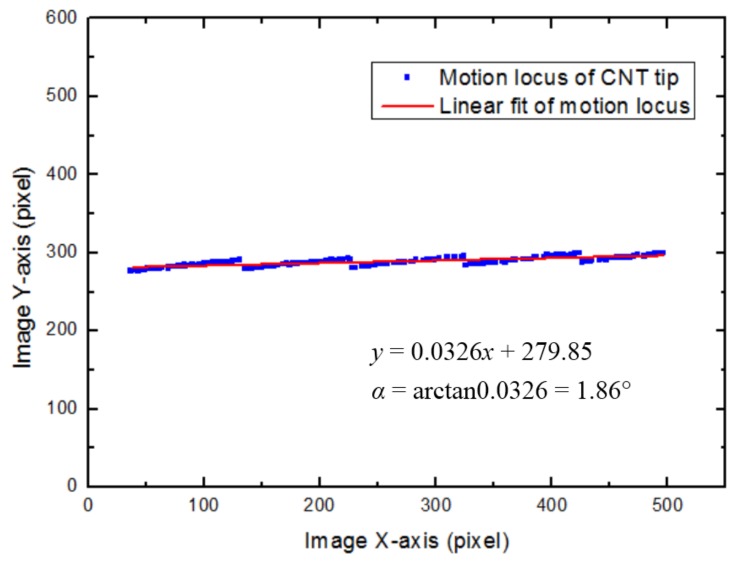
Recording the motion locus of CNT tip vision-based tracking for X-axis, SEM magnification was 15,000×, the blue dots were the tracking points, and the red line was their linear fit.

In our system, the motion trajectory of each manipulator along the X/Y-axis was determined by visually tracking the distal CNT tip in real time. Figure 11 demonstrates the motion trajectory of the X-axis nanomanipulator at an image magnification of 15,000×. The rotation angle, *α*, between the manipulator’s X-axis and the image’s X-axis can be calculated by fitting this trajectory line. Using the same method, the angle, *β*, can be determined.

## 4. Experiments and Discussion

**Figure 12:**
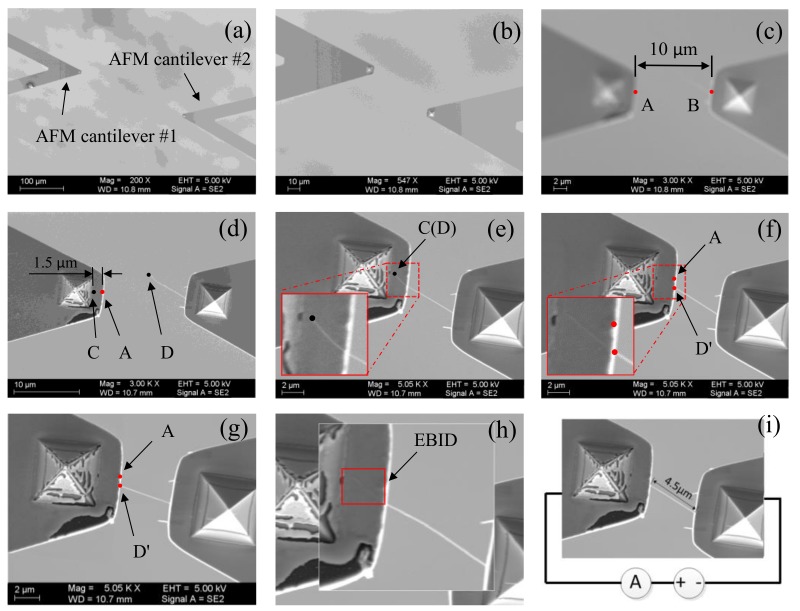
The procedure of automated measurement of individual CNTs by visual recognition. (**a**) Moving the two AFM cantilevers toward he FOV center for their initial positions; (**b**,**c**) Automatically moving the two AFM cantilevers toward the FOV center using the magnification-regulated speed adapting approach; (**d**) Coarse positioning of AFM cantilever #1 and AFM cantilever #2 based on the sharpness depth estimation method; (**e**) Achieving an overlap (1μm) between AFM cantilever #1 and the CNT; (**f**,**g**) Contact detection between AFM cantilever #1 and the CNT; (**h**) Fixing the CNT on AFM cantilever #1 via deposition electron-beam include deposition (EBID) soldering; (**i**) Measuring the CNT’s electrical conductivity.

In order to verify the feasibility of the above-presented methods, an automated nanomanipulation experiment was implemented to perform electrical characterization of individual CNT based on visual feedback. The operational workflow is illustrated in Figure 12.

The two manipulators were firstly controlled to move AFM cantilever #1 and AFM cantilever #2 into the SEM FOV under a low magnification of 300× in a manual control manner, as shown in Figure 12a. By comparing the barycenter locations of the both AFM cantilevers contours, they were distinguished from each other. As the SEM magnification increased, the proposed visual feedback control, based on the magnification-regulated speed adapting approach proposed in Section 3.3, was implemented to automatically move the two manipulators towards the FOV center. Once the distance between the two AFM cantilever tips was 10 μm, the CNT tip could be accurately recognized at a magnification of around 3000×, as shown in Figure 12b,c. Then, both AFM cantilevers stopped moving, and the automated centering operation took about 18 s. Next, both cantilevers were placed in the focal plane based on the proposed sharpness-based depth estimation method, followed by moving AFM cantilever #1 downward 3μm along the Z-direction. This ensured that the used CNT was vertically above the AFM cantilever #1 for further contact detection, as shown in Figure 12d. This procedure applied coarse positioning and took less than 25 s.

To complete the contact detection between AFM cantilever #1 and the CNT, AFM cantilever #1 was visually controlled in the XY-plane to approach the CNT tip from underneath, until point C coincided with the CNT tip (point D), as shown in Figure 12e. This process took 5 s. Point C was selected on the left of point A with a distance of 1.5 μm to ensure an overlapping length of more than 1 μm between AFM cantilever #1 and the CNT. Then, AFM cantilever #1 was moved at a constant Z-direction speed of 100 nm/s to approach the CNT, taking about 45 s. During this ascending process, the visual system did not acquire or monitor the actual vertical distance between AFM cantilever #1 and the CNT in real-time. When AFM cantilever #1 moved close to the CNT at a distance of around 1.5 μm, the free end of the CNT abruptly deflected downward and adhered on the surface of AFM cantilever #1. This happened because of the van der Waals forces between AFM cantilever #1 and the CNT and caused the CNT tip (point D) to contact point D’, as shown in Figure 12f. According to this phenomenon, visually tracking the CNT’s tip position can estimate the contact state between AFM cantilever #1 and the CNT. Until the displacement (D_DD’_) between point D and point D’ surpassed a threshold value (~0.5 μm), the Z-direction movement of AFM cantilever #1 was halted.

To ensure complete and stable contact, AFM cantilever #1 was controlled to move 0.5 μm along the Y direction, as shown in Figure 12g. If the relative position between point A and point D’ was constant, their contact status could be determined as stable. On the contrary, if their contact was not stable, the system would return to the position of AFM cantilever #1 and the CNT in the XY-plane and perform the contact detection in the Z-direction again. After completing stable contact, as shown in Figure 12h, the CNT needed to be soldered and fixed on AFM cantilever #1 by electron-beam include deposition (EBID) [25]. This soldering contributed to reducing the contact resistance between AFM cantilever #1 and the CNT dramatically. Finally, a semiconductor characterization system (Keithley 4200-SCS, Tektronix, OH, USA) was connected to the two AFM cantilevers for measuring the CNT’s electrical conductivity, as shown in Figure 12i.

**Figure 13:**
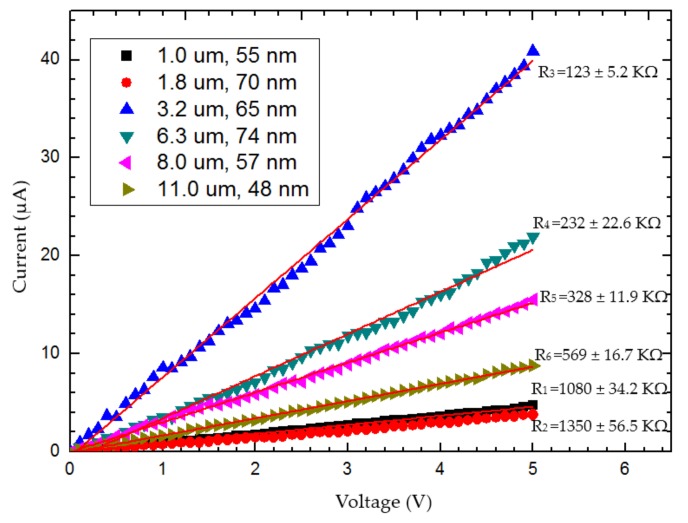
I-V characteristics of the measured CNTs.

To quantify the repeatability of the above proposed technique, the entire automation contact procedure of the CNT was tried 30 times on six prepared CNTs that adhered to AFM cantilevers; the CNTs varied in protruding orientation, length (1–11 μm) and diameter (48–74 nm), thus representing different circumstances of automatic manipulation in terms of orientation and flexibility. For each of the six CNTs, the contact procedure was attempted five times on the CNT before the final EBID soldering. Each time after AFM cantilever #1 contacted the CNT from below and was ready for EBID, it was retracted and brought to a different starting XYZ position for the next trial. Once the fifth trial of contact operation had been accomplished, the CNT was fixed on AFM cantilever #1 by EBID-soldering, then electrified to measure its electrical characterization; the results of the six CNTs’ I-V data are shown in Figure 13.

The system successfully completed 30 trials (process without EBID), resulting in a 100% repeatability that indicates the feasibility and reliability of the proposed methods in this paper. Assessment of the time taken for each trial showed that the shorter CNTs took more time (the 1 μm CNT took around 55 s) in regard to the CNT contact detection process than the longer CNTs (the 11 μm took cost around 35 s). In addition, the six CNTs’ resistance values can be calculated via their respective I-V data, and the results show that two of them (R_1_ and R_2_) were markedly greater than the other four CNTs resistance values, as shown in Figure 13, which indicates that the measurement results of the shortest two CNTs included larger amounts of error. This issue may be related to the length of the CNTs. According to geometric features of AFM cantilevers and CNTs, the contact situations between AFM cantilever #1 and the CNTs were divided into two types: point (or extremely short lines) contact and linear contact. Point contact happened when only the CNT tip contacted AFM cantilever #1, whereas linear contact means that the overlapping portion between AFM cantilever #1 and the CNT was in full contact.

For the issues mentioned above, we speculate that longer CNTs are more likely to be drawn down and compliantly adhere to AFM cantilever #1 because of their greater flexibility. Hence, longer CNTs were more likely to show linear contact, and the contact occurred earlier, even though the distance between AFM cantilever #1 and the CNT in the Z-direction was still far. Conversely, shorter CNTs are more likely to show point contact and consume more time in the process of automatic contact detection. Compared with linear contact, point (or extremely short lines) contact generates a large contact resistance between AFM cantilever #1 and a CNT [26]; this is why the shortest CNTs (the 1 μm CNT and the 1.8 μm CNT) had higher resistance values than the other four CNTs. In order to avoid the situation of point contact, a dynamic detection method has been proposed in the literature [27,28] that can recognize the two contact types and adjust point contact to linear contact automatically. Further research will involve the use of this method to solve the issue of CNT contact (this paper did not cover details of the method).

The CNT resistivity was determined by calculating the average value of the four resistivity values of CNT (linear contact) which was (1.19 ± 0.196) × $10^{-4}$ Ω·m; the major error in this result resulted from the imprecise measurement of CNT length and diameter in the SEM image. The entire process of automated measurement took approximately 2 min on average (without considering the time for EBID), whereas a skilled human operation needs at least 8 min to observe the SEM imaging and adjust nanomanipulators. Compared with manual operation, automatically measuring the electrical characterization of a CNT is of great significance to nanorobotic techniques, because it not only saves time in the batch measurement of CNTs, but also reduces the operating difficulty and avoids undesired destruction of specimens and nanomanipulators due to man-made mistakes.

## 5. Conclusions

A visual servoing-based nanorobotics manipulation system was developed for automated 3D-nanomanipulation of nanometer-scale objects. This system maintained higher positioning accuracy, owing to precise recognition of targets and precise control of manipulators, and higher working efficiency during the centering operation of end-effectors, via a proposed magnification-regulated speed adapting approach. Moreover, in order to realize efficient and precise positioning in the Z-direction, a coarse-to-fine positioning method, combining sharpness-based depth estimation and contact detection was applied. The experimental results indicated the efficiency and stability of the system for automated measurement of electrical characterization for individual CNTs. In the future, we will improve this system further by focusing on different target objects’ recognition and intelligent control to expand the system’s functionality for meeting different nanomanipulation tasks.

## Supplementary Materials

The following are available online at http://www.mdpi.com/1424-8220/18/4/1137/s1, Figure S1. The procedure of automated measurement of individual CNT by visual recognition. Group 1 CNT with length: 1.0 μm, diameter: 55 nm. Figure S2. The procedure of automated measurement of individual CNT by visual recognition. Group 1 CNT with length: 1.8 μm, diameter: 70 nm. Figure S3. The procedure of automated measurement of individual CNT by visual recognition. Group 1 CNT with length: 3.2 μm, diameter：65 nm. Figure S4. The procedure of automated measurement of individual CNT by visual recognition. Group 1 CNT with length: 6.3 μm, diameter: 74 nm. Figure S5. The procedure of automated measurement of individual CNT by visual recognition. Group 1 CNT with length: 8.0 μm, diameter: 57 nm. Figure S6. The procedure of automated measurement of individual CNT by visual recognition. Group 1 CNT with length: 11 μm, diameter: 48 nm. Figure S7. I-V curve of 6 groups of CNTs. Table S1: Resistance and resistivity of 6 groups of CNTs.
