# Supplementary material for: Visual Servoing-Based Nanorobotic System for Automated Electrical Characterization of Nanotubes inside SEM
Source: Sensors (Basel). 2018 Apr 8;18(4):1137. doi: 10.3390/s18041137 (PMC5948737; doi:10.3390/s18041137)
Supplement: Supplementary file 1 [file sensors-18-01137-s001.pdf]

sensors

MDPI

Supplementary Materials

6. Groups of different CNTs Experiments

Length: 1.0μm, Diameter：55 nm.

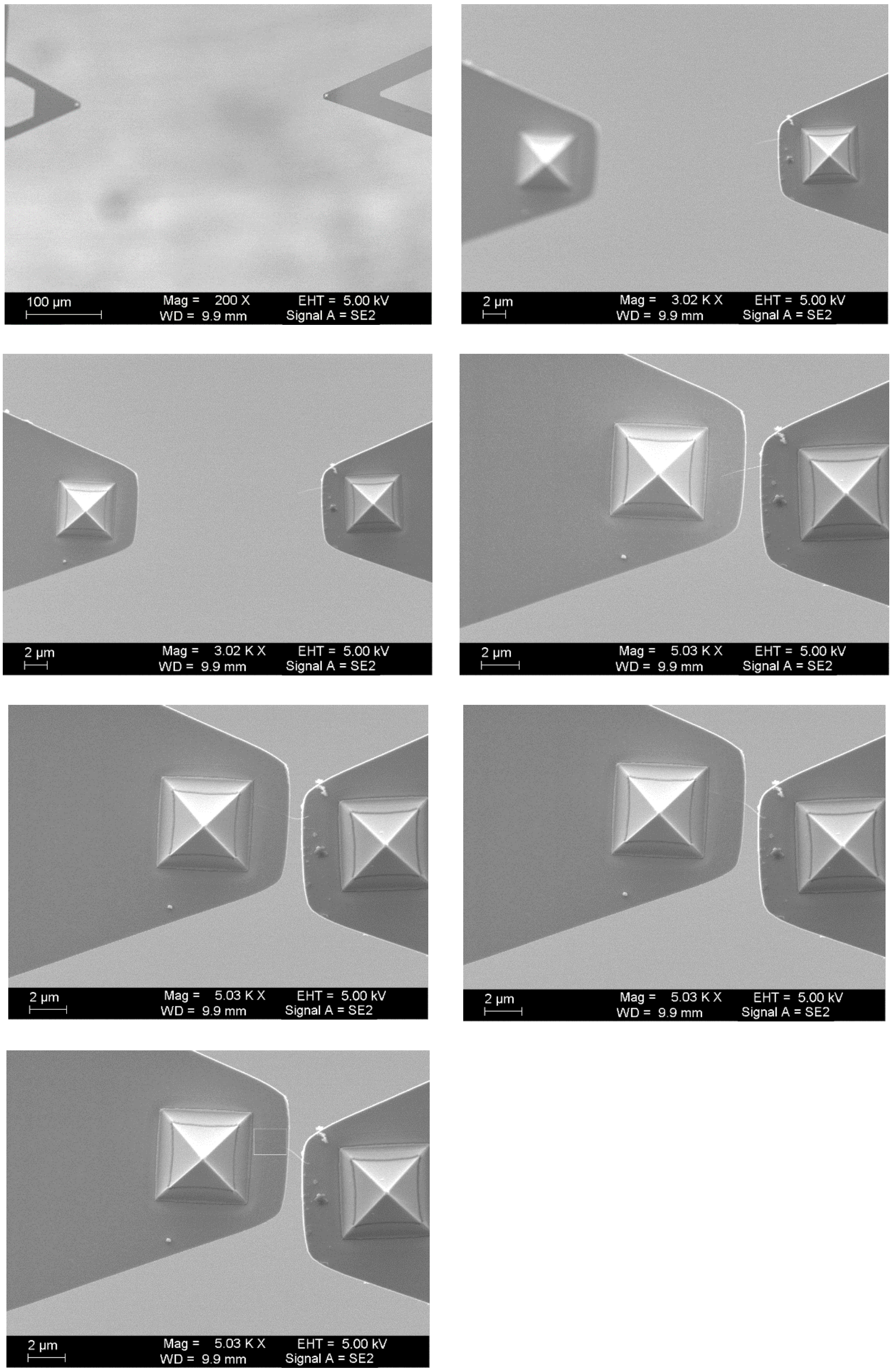

Figure S1. The procedure of automated measurement of individual CNT by visual recognition. Group 1 CNTwith length: 1.0μm, diameter：55 nm.

sensors

MDPI

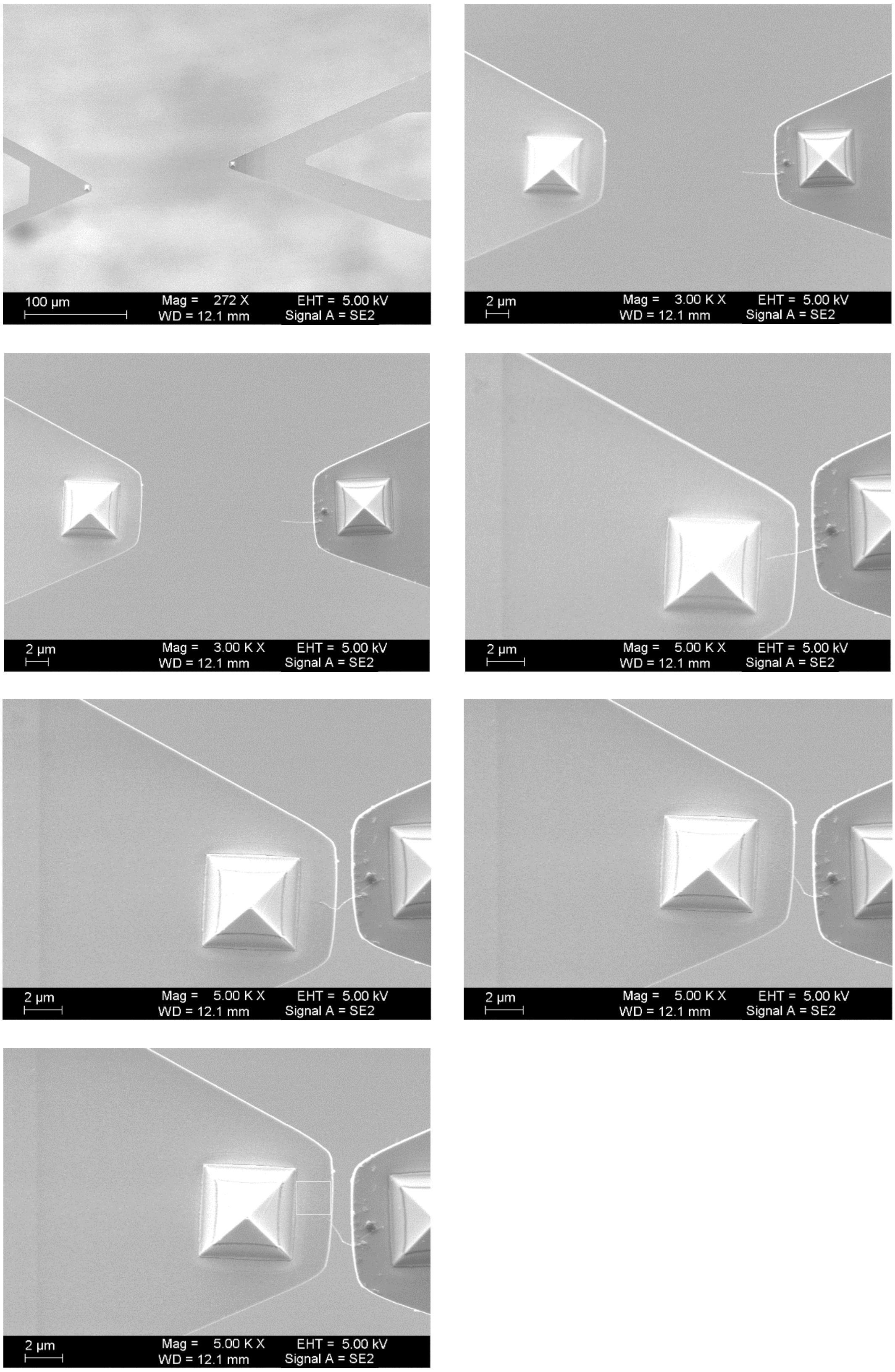

Figure S2. The procedure of automated measurement of individual CNT by visual recognition. Group 1 CNT with length: 1.8µm, diameter：70 nm.

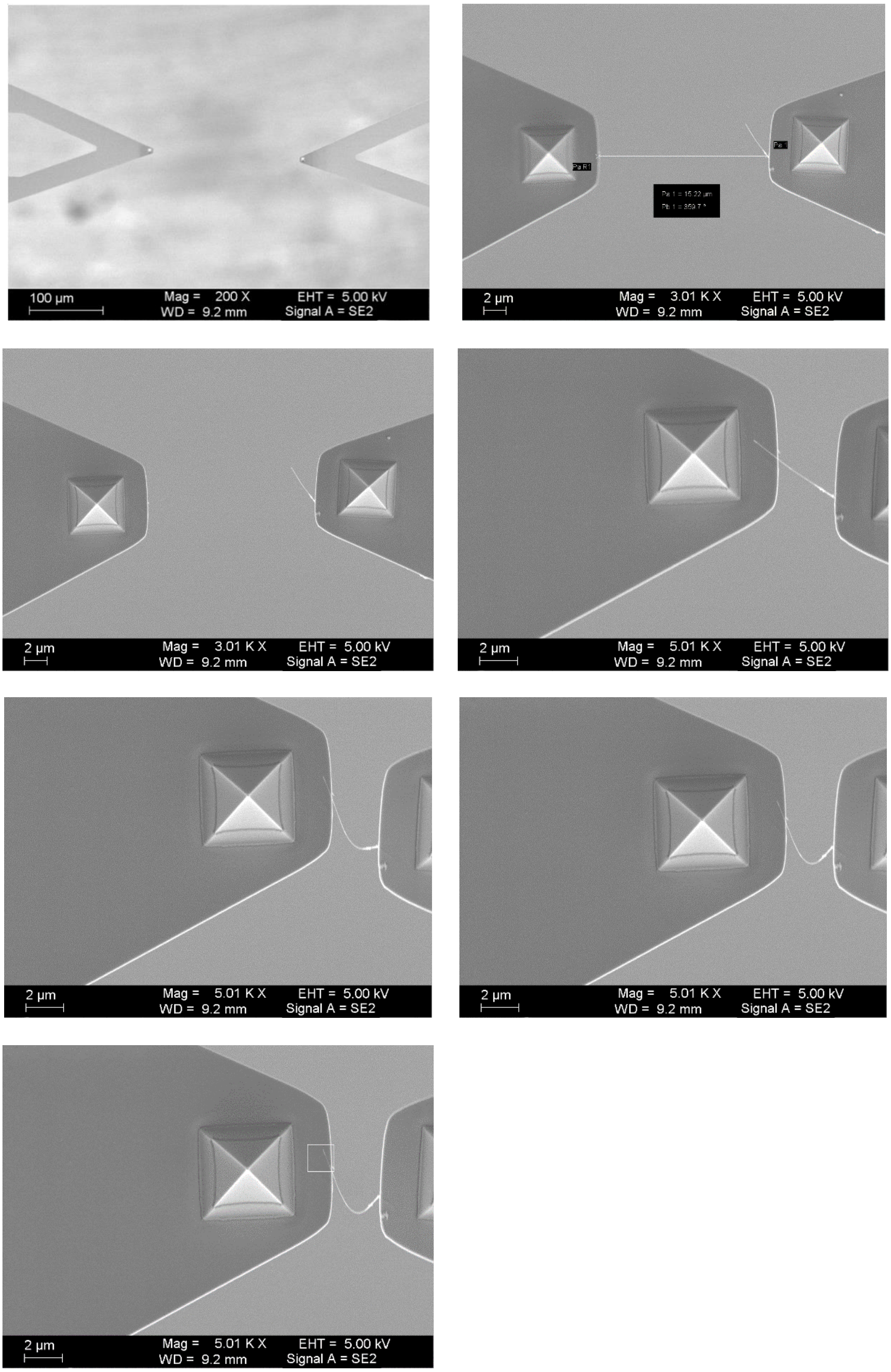

Figure S3. The procedure of automated measurement of individual CNT by visual recognition. Group 1 CNT with length: 3.2μm, diameter： 65 nm.

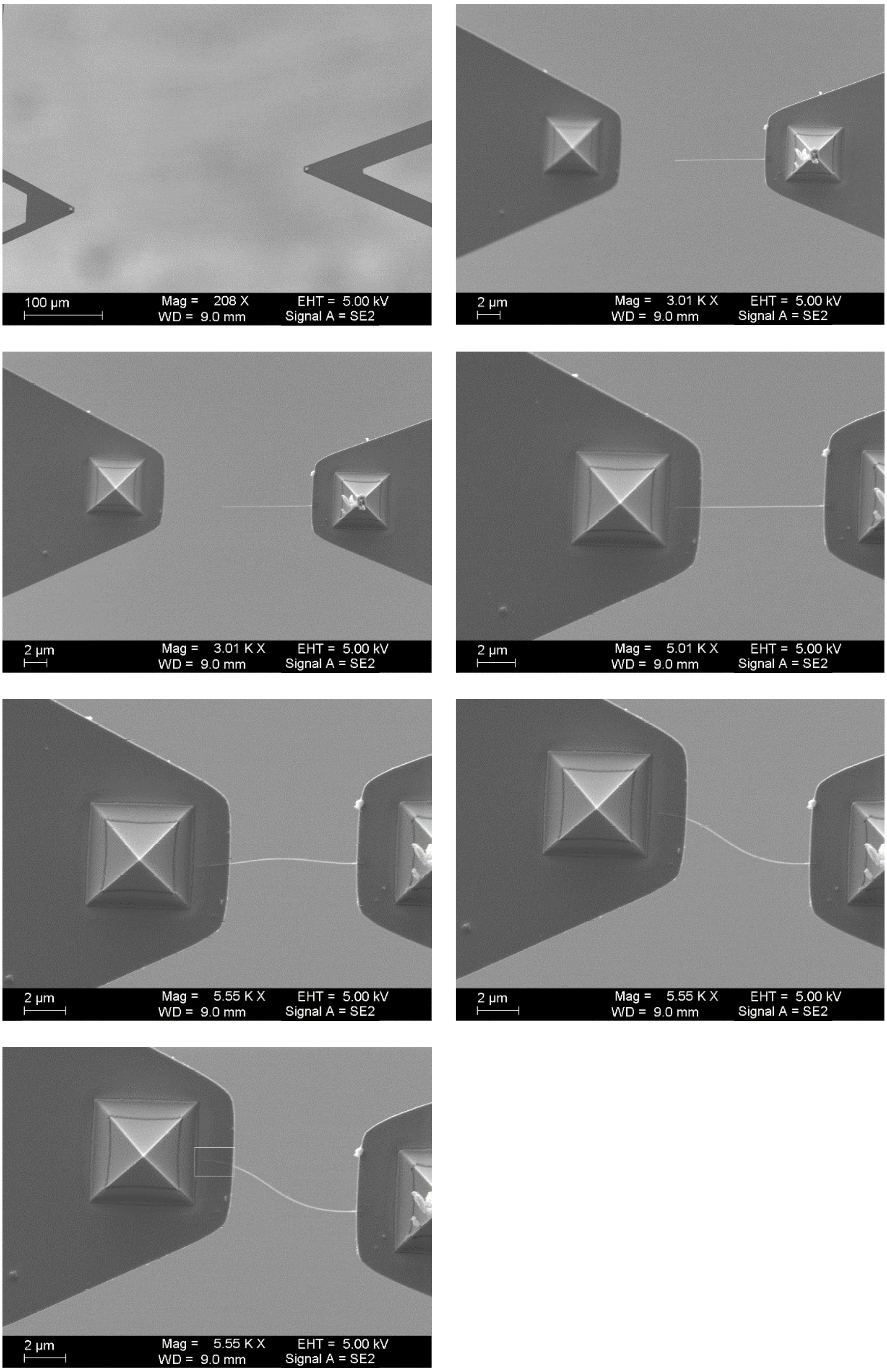

Figure S4. The procedure of automated measurement of individual CNT by visual recognition. Group 1 CNT with length: 6.3μm, diameter：74 nm.

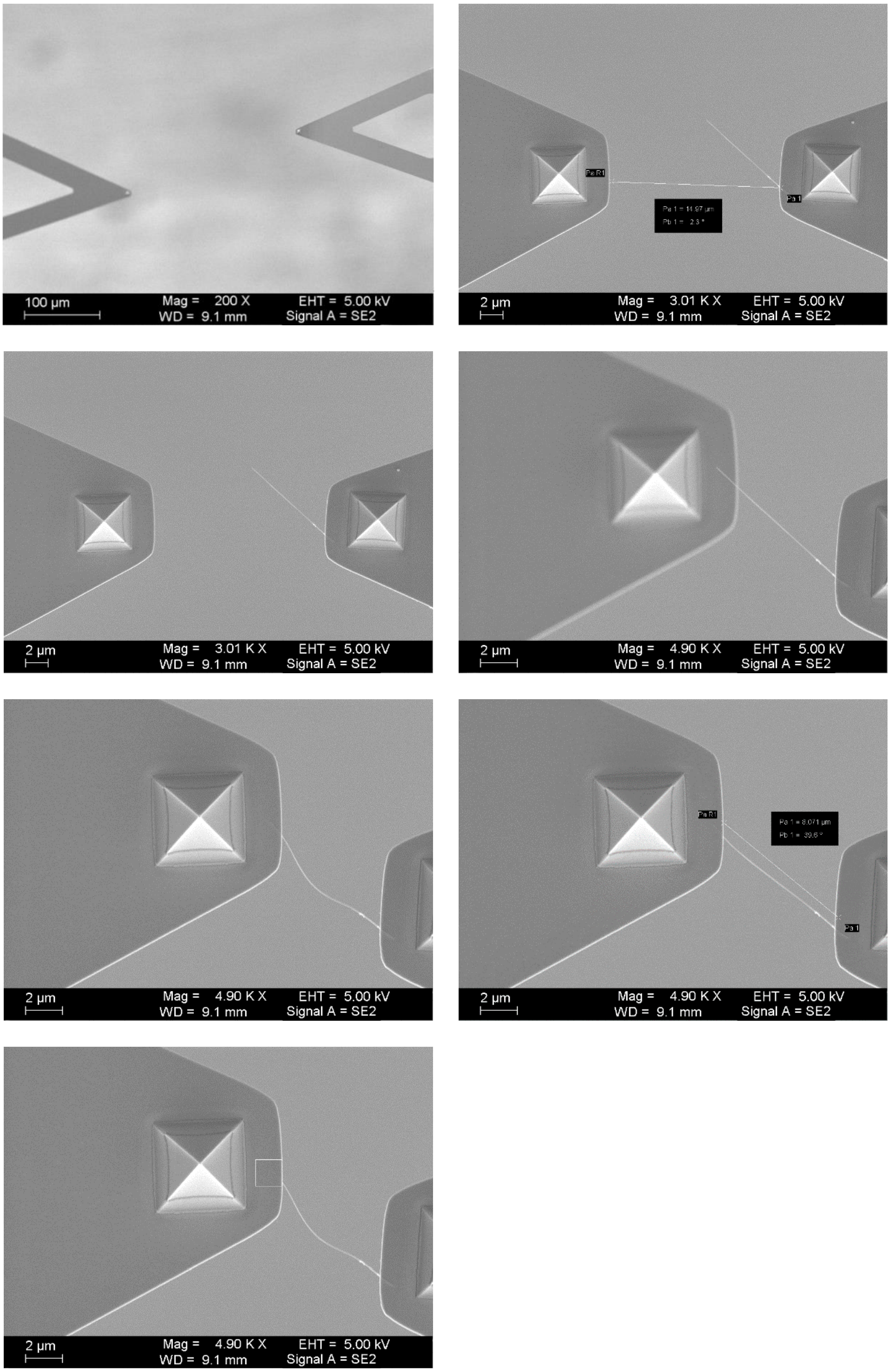

Figure S5. The procedure of automated measurement of individual CNT by visual recognition. Group 1 CNT with length: 8.0μm, diameter：57 nm.

sensors

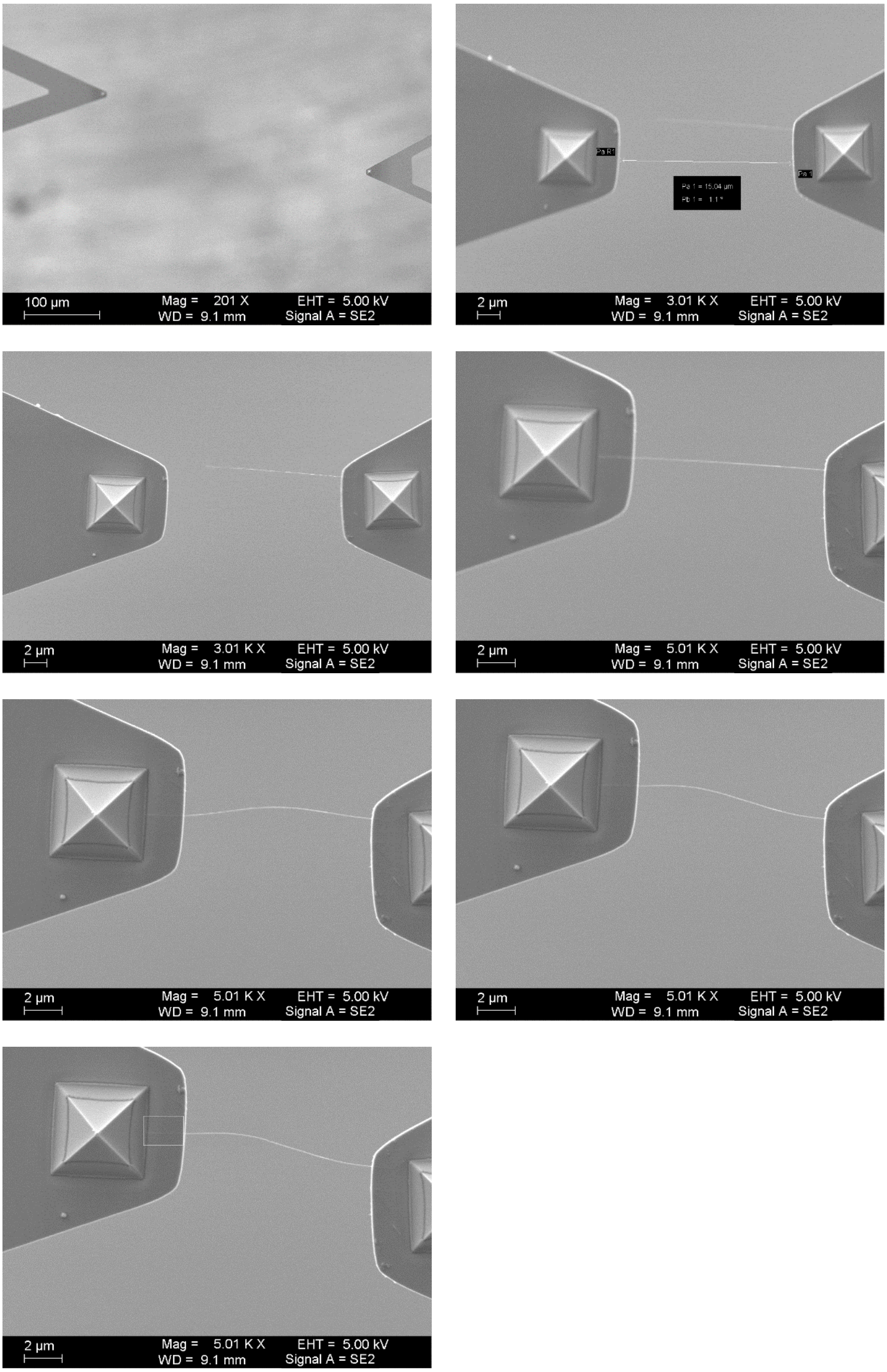

Figure S6. The procedure of automated measurement of individual CNT by visual recognition. Group 1 CNT with length: 11μm, diameter : 48 nm.

sensors

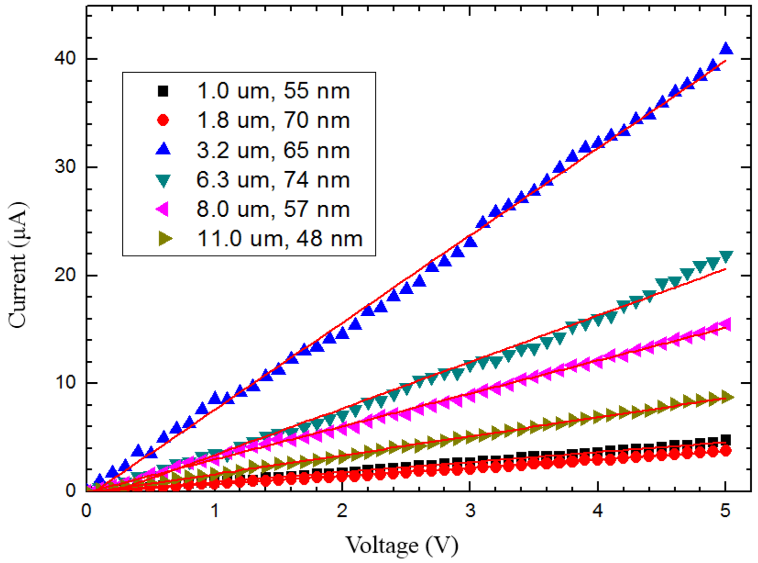

Figure S7. I-V curve of 6 groups of CNTs.

Table S1. Resistance and resistivity of 6 groups of CNTs.

**CNT size**	**1.0μm**	**1.8μm**	**3.2μm**	**6.3μm**	**8.0μm**	**11.0μm**
Resistance (KΩ)	1080 ± 34.2	1350 ± 56.5	123 ± 5.2	232 ± 22.6	328 ± 11.9	569 ± 16.7
Resistivity (Ω·m)	2.57×10^{-3}	5.18×10^{-3}	1.18×10^{-4}	1.58×10^{-4}	1.05×10^{-4}	0.94×10^{-4}

© 2018 by the authors. Submitted for possible open access publication under the terms and conditions of the Creative Commons Attribution (CC BY) license (http://creativecommons.org/licenses/by/4.0/).
